# PPARs and Liver Disease

**DOI:** 10.1155/2013/896412

**Published:** 2013-02-05

**Authors:** Yasuteru Kondo, Kenji Uno, Keigo Machida, Masanori Terajima

**Affiliations:** ^1^Division of Gastroenterology, Tohoku University Graduate School of Medicine, 1-1 Seiryo, Aoba, Sendai, Miyagi 980-8574, Japan; ^2^Division of Molecular Metabolism and Diabetes, Tohoku University Graduate School of Medicine, 1-1 Seiryo, Aoba, Sendai, Miyagi 980-8574, Japan; ^3^Department of Molecular Microbiology and Immunology, Keck School of Medicine, University Southern California, Los Angeles, CA 90033, USA; ^4^Division of Infectious Diseases and Immunology, Department of Medicine, University of Massachusetts Medical School, 55 Lake Avenue North, Worcester, MA 01655, USA

This special issue of PPAR Research contains four interesting reviews and a research article examining the relevance of PPARs to liver diseases. Peroxisome proliferation-activated receptors (PPARs) are members of the nuclear hormone receptor superfamily and have been implicated in a variety of pathologic processes. PPARs require heterodimerization with retinoid X receptors (RXRs) to function. PPARs *α*/*β*/*δ*/*γ*, with RXR, are important nuclear receptors expressed in the liver and contribute to the control of glucose and lipid metabolism, cell proliferation and inflammation, and so forth. PPARs were considered target molecules of human metabolic disease such as nonalcoholic fatty liver diseases (NAFLDs) including nonalcoholic steatohepatitis (NASH), a condition that might progress to cirrhosis. In this special issue, two review articles mention the relationship between PPARs and NAFLD. In regard to inflammation, a review article summarizes the antioxidant stress and anti-inflammation of PPAR*α*. On the other hand, a research article mentions that PPAR*γ* exacerbated concanavalin A (Con A)-induced liver injury. In our review article, we summarize the relevance of PPARs and hepatocellular carcinoma (HCC) including cancer stem cells. These five articles have interesting and valuable points of views regarding PPARs and liver diseases.

In the review article “Antioxidant stress and anti-inflammation of PPAR*α* on warm hepatic ischemic-reperfusion injury” by Z. Gao and Y. H. Li, the authors focus on hepatic ischemic-reperfusion injury, since PPAR*α* could have a role in organ protection in addition to regulating lipid and lipoprotein metabolism. They concluded that oxidant stress and inflammation are the most critical mechanisms in organ pathophysiology after warm hepatic ischemia reperfusion. The most significant mechanisms of PPAR*α* hepatoprotective abilities have been demonstrated through antioxidant stress and anti-inflammation functions. Moreover, they mention that PPAR*α* agonists such as N-3 polyunsaturated fatty acids, eicosapentaenoic acid, and docosahexaenoic acid could decrease the expression of proinflammatory genes by preventing I*κ*B phosphorylation and NF-*κ*B translocation into the nucleus. On the other hand, Y. Ogawa et al. published the research article “*Peroxisome proliferation-activated receptor gamma exacerbates concanavalin A-induced liver injury via suppressing the translocation of NF-*κ*B into the nucleus*.” Using a mice model, this article surprisingly shows that the administration of PPAR*γ* ligands exacerbated Con A-induced liver injury. They concluded that PPAR*γ* suppressed the translocation of NF-*κ*B into the nucleus, thereby inhibiting the suppression of liver cell apoptosis. In clinical settings, liver damage would occur with inflammation and apoptosis. Therefore, we need to consider the opposite effects of PPARs on liver injury.

NAFLD, a major cause of progressive liver disease, is increasing worldwide at an alarming rate. Defined by an increased hepatic lipid content, NAFLD varies widely from simple steatosis to NASH and has a strong genetic component. PPARs, including PPAR*α*, PPAR*γ*, and PPAR*δ*, play an important role in hepatic lipid metabolism and also have several genetic variants (polymorphisms). In the review article “*Peroxisome proliferator-activated receptor genetic polymorphisms and nonalcoholic fatty liver disease: any role in disease susceptibility?*” by P. Dongiovanni et al., the authors conducted a meta-analysis of previously reported evidence based upon which they describe the possible association between PPARs genetic polymorphisms and the susceptibility to NAFLD and NASH in specific subgroups. This review may contribute to new insight into the management of a therapeutic strategy for NAFLD, targeting PPARs. In the review article “*Misregulation of PPAR functioning and Its pathogenic consequences associated with nonalcoholic fatty liver disease in human obesity*” by L. A. Videla and P. Pettinelli, the authors mention that NASH is involved in the misregulation of PPARs signaling, accompanied by PPAR-*γ* and SREBP-1c-mediated metabolic disturbances (obesity-induced oxidative stress and related long-chain polyunsaturated fatty acid n-3 (LCPUFA n-3) depletion, insulin resistance, hypoadiponectinemia, and ER stress, due to lipogenesis and fatty acid oxidation. Targeting PPAR-*α* is problematic since fibrates have poor effectiveness, thiazolidinediones have weight gain limitations, and dual PPAR-*α*/*γ* agonists have safety concerns. The authors describe that supplementation of LCPUFA n-3 is a novel therapeutic modality since it reduces liver steatosis scores and inflammatory response, since the LCPUFA n-3 depletion reduces PPAR-*α*, leading to enhanced DNA binding of proinflammatory factors (NF-*κ*B and AP-1) and the progression of steatosis to steatohepatitis.

In our review article “PPAR could contribute to the pathogenesis of hepatocellular carcinoma,” we summarize the relevance of PPARs to the pathogenesis of HCC and cancer stem cells and possible therapeutic options through modifying PPAR signaling, since PPARs could contribute to the mechanisms of cell cycling, anti-inflammatory responses, and apoptosis. Abnormal stimulation of PPAR*α* generates HCC through fatty liver. In HCCs, it is not clear whether PPAR*γ* promotes cancer or can control it. PPARs might be useful target cancer stem cells in inducing the differentiation of HCC, because the expression of PPARs has been implicated in the regulation of the cell cycling of hepatocytes.

In conclusion, we hope that you will find these recent advances in elucidating the roles of PPARs in the various kinds of liver diseases. We expect that the reviews presented in this special issue, on the interplay between PPARs and liver disease, will be highly useful for those with interest in this field.

*Yasuteru Kondo* *Yasuteru Kondo* *Kenji Uno* *Kenji Uno* *Keigo Machida* *Keigo Machida* *Masanori Terajima* *Masanori Terajima*

